# FimH and Anti-Adhesive Therapeutics: A Disarming Strategy Against Uropathogens

**DOI:** 10.3390/antibiotics9070397

**Published:** 2020-07-10

**Authors:** Meysam Sarshar, Payam Behzadi, Cecilia Ambrosi, Carlo Zagaglia, Anna Teresa Palamara, Daniela Scribano

**Affiliations:** 1 Department of Public Health and Infectious Diseases, Sapienza University of Rome, Laboratory affiliated to Institute Pasteur Italia- Cenci Bolognetti Foundation, 00185 Rome, Italy; meysam.sarshar@uniroma1.it (M.S.); annateresa.palamara@uniroma1.it (A.T.P.); 2 Research Laboratories, Bambino Gesù Children’s Hospital, IRCCS, 00146 Rome, Italy; 3 Microbiology Research Center (MRC), Pasteur Institute of Iran, Tehran 1316943551, Iran; 4 Department of Microbiology, College of Basic Sciences, Shahr-e-Qods Branch, Islamic Azad University, Tehran 37541-374, Iran; 5 IRCCS San Raffaele Pisana, Department of Human Sciences and Promotion of the Quality of Life, San Raffaele Roma Open University, 00166 Rome, Italy; 6 Department of Public Health and Infectious Diseases, Sapienza University of Rome, 00185 Rome, Italy; carlo.zagaglia@uniroma1.it (C.Z.); ; daniela.scribano@uniroma1.it (D.S.); 7 Dani Di Giò Foundation-Onlus, 00193 Rome, Italy

**Keywords:** FimH, adhesins, uropathogenic *Escherichia coli*, uropathogenic *Klebsiella pneumoniae*, uropathogenic *Proteus mirabilis*, urinary tract infection, antagonists, mannose-binding lectin, affinity

## Abstract

Chaperone-usher fimbrial adhesins are powerful weapons against the uropathogens that allow the establishment of urinary tract infections (UTIs). As the antibiotic therapeutic strategy has become less effective in the treatment of uropathogen-related UTIs, the anti-adhesive molecules active against fimbrial adhesins, key determinants of urovirulence, are attractive alternatives. The best-characterized bacterial adhesin is FimH, produced by uropathogenic *Escherichia coli* (UPEC). Hence, a number of high-affinity mono- and polyvalent mannose-based FimH antagonists, characterized by different bioavailabilities, have been reported. Given that antagonist affinities are firmly associated with the functional heterogeneities of different FimH variants, several FimH inhibitors have been developed using ligand-drug discovery strategies to generate high-affinity molecules for successful anti-adhesion therapy. As clinical trials have shown d-mannose’s efficacy in UTIs prevention, it is supposed that mannosides could be a first-in-class strategy not only for UTIs, but also to combat other Gram-negative bacterial infections. Therefore, the current review discusses valuable and effective FimH anti-adhesive molecules active against UTIs, from design and synthesis to in vitro and in vivo evaluations.

## 1. Introduction

Urinary tract infections (UTIs) caused by different microbial agents, including both Gram-positive and Gram-negative bacteria and fungi, are among the most prevalent infectious diseases affecting millions of people annually [1,2,3,4,5,6]. Most UTIs are community- and nosocomial-acquired infections, which can occur in any part of the urinary tract [4,6,7,8,9,10]. Among the Gram-negative bacteria, the uropathogenic *Escherichia coli* (UPEC) accounts for 50% of nosocomial- and up to 95% of community-acquired UTIs, followed by the uropathogenic *Klebsiella pneumoniae* (UPKP) and the uropathogenic *Proteus mirabilis* (UPPM) [1,6,11,12]. Although UTIs can occur in both male and female patients, the number of female patients is significantly higher than male patients due to the anatomical features of women. These features include the shortness of the urethra, the urethral meatus’s proximity to the anus, and the more humid surrounding environment compared to the male anatomy [1,4,5,6,10]. Despite receiving appropriate antibiotic therapy, 20–30% of women who have already experienced an initial UTI develop a recurrent infection (rUTI) within 4–6 months. The high recurrence rates of UTIs, and the increasing antimicrobial resistance among uropathogens, impose a significant economic burden. As such, the inefficacy of antibiotic therapies determines the urgent need for the development of alternative strategies for UTIs [1,13,14]. In this regard, alternative non-antibiotic strategies that target uropathogenic adhesive factors are highly attractive. Indeed, adhesion to host cells represents a critical step in the early stages of infection, allowing bacteria to contact, colonize and, eventually, establish the infection. Thus, uropathogens have developed a plethora of adhesive structures that enable bacterial colonization of the urinary tract [5,6,8,13,15,16]. The three most common uropathogenic pathogens, UPEC, UPKP and UPPM, are armed by adhesive pili or fimbriae, mostly belonging to the Chaperone-usher (CU) pathway [5,12]. The axial and pivotal role of these important virulence factors in bacterial pathogenicity and virulence has sped up the development of anti-fimbrial therapeutic approaches [17]. Among these new approaches, the anti-adhesive strategy seems an effective and valid treatment procedure for UTIs. Detailed structural characterization of these adhesive organelles, as well as their receptors and ligands, may help us to find new antagonists to compete with adhesins. However, lab costs have reduced the speed of success in this regard. Fortunately, in recent years, computational biology and chemistry, bioinformatics, and different databases and software tools have hastened the achievements in this field. To prepare this review, the authors used the following MeSH keywords: FimH, adhesins, uropathogenic *Escherichia coli*, urinary tract infections, FimH antagonists, mannose-binding lectin, α-mannoside. This highlighted the great advances that have been obtained in UPEC FimH anti-adhesive antagonists from 1977 up to now. Therefore, the current review discusses valuable and effective FimH anti-adhesive molecules against UTIs, from their design and synthesis to in vitro and in vivo evaluations.

## 2. The Importance of Anti-Adhesive Strategy 

The use of antimicrobial agents for the treatment of different infectious diseases like UTIs started more than eight decades ago. However, the number of antibiotic-resistant strains arose soon after antibiotic introduction, leading to the worldwide spread of multi- and pan-drug-resistant strains (MDR and PDR, respectively), also known as superbugs [18,19,20,21]. Antimicrobial resistance (AMR) is recognized as one of the major global health challenges of the 21st century, with 10,000,000 estimated deaths per year by 2050, and heavy economic consequences for public health and governments [20,22]. Among the species causing UTIs, UPEC, UPKP and, to a lesser extent, UPPM are considered the most worrisome bacteria for AMR. The diffuse practice of prescribing antibiotics to treat UTIs without any bacterial characterization, together with nosocomial MDR strains, has increased significantly the rates of MDR uropathogens, thereby leading to ineffective antibiotic therapies and the persistence of these microorganisms in health care facilities [23]. In Europe, the AMR of uropathogens is deeply detailed by the surveillance system of the European Center of Diseases Prevention and Control (ECDC) (https://www.ecdc.europa.eu/en/about-us/networks/disease-networks-and-laboratory-networks/ears-net-data). To counteract AMR, several alternative therapeutic approaches are emerging, including anti-adhesive strategies [4,6,10,13,24]. Uropathogens belonging to the *Enterobacteriaceae* family share a huge number of virulence factors known to be involved in the adhesion, colonization and invasion of host tissues, offering the possibility to develop different and specifically targeted anti-adhesive strategies. Dissecting the mechanisms by which uropathogens establish an infection, the molecules involved in the primary host–pathogen interaction appeared to be the most relevant virulence factors for urothelium colonization. Indeed, uropathogens express cell surface structures, including outer membrane proteins, secretion systems, appendages and specific adhesins, to promote tissue adhesion. The adhesion phase is the first step of colonization, and anti-adhesive strategies represent an effective mechanism for inhibiting the following phases of infection [18,19,21,25]. Among surface structures, adhesins are specialized bacterial proteinaceous adhesive molecules with defensive and offensive characteristics, able to interact with both the peptidic and the glycosylic residues present on the eukaryotic cell surface and in the extracellular matrix. Thus, the ability to bind to different host structures guarantees the success of bacterial attachment [26,27]. Adhesins can be distinguished according to their molecular structure in adhesive appendages and adhesive molecules. Adhesive appendages are multimeric complexes formed by different proteins, resulting in visible bacterial organelle, such as pili or fimbriae [28]. Although the entire structure is needed for full functionality, the tips of these structures often contain the actual adhesin, which binds to a host receptor. On the other hand, an adhesive molecule is a stand-alone surface protein, most commonly attached to the outer membrane or cell wall, the intrinsic structure of which facilitates the binding to a host receptor [28]. Adhesins are also involved in biofilm formation, antimicrobial resistance, and in the mechanism by which bacteria can be internalized within cells [25,26,27,29]. Hence, blocking the bacterial adhesins is the most preferred strategy for keeping the hosts safe from UTIs [30]. 

## 3. Treasure of Chaperone-Usher Adhesins

The CU pathway is a conserved protein secretion system, able to assemble a diverse array of pili on bacterial surfaces. These pili, known also as fimbriae or fibrillae, represent pivotal urovirulence factors in Gram-negative bacteria and, in particular, in *Enterobacteriaceae* family members, e.g., UPEC, UPKP and UPPM. Phylogenetic studies on the CU system revealed 38 different CU fimbriae in *E. coli*, encoded by 458 chromosomal CU operons [31,32]. It is reported that more than 60% of UPEC strains carry 5 to 15 CU fimbriae [33]. Type 1, type 3, type 9, S, P, F1C and Auf are the most common CU fibers among UPEC pathotypes [5,31]. The first characterized virulence adhesive factors of UPEC were the type P fimbriae, encoded by the *pap* (pyelonephritis-associated pili) genes, which are significantly prevalent among the strains of UPEC that cause pyelonephritis [13]. This pilus is structurally similar to type 1 pili, and exhibits the adhesin PapG at its tip. Importantly, among UPEC pathotypes, three different alleles for the PapG adhesin, including PapGI, PapGII and PapGIII, have been recognized, with class II being the allele predominantly associated with human pyelonephritis, and class III correlated with human cystitis [5,34]. Different from type 1 pili, it is also known as a mannose-resistant pilus, because it does not interact with mannose sugar residues but with galabiose saccharide residues. The differential distribution of the receptor isotypes in different hosts and tissues and the binding specificity of the various PapG adhesins account for the host and tissue tropisms of UPEC. Moreover, the expression of both type 1 and Pap pili enables bacteria to bind to bladder and kidney cells expressing mannosylated uroplakins and galabiose rich sphingolipids, respectively [34,35].

*P. mirabilis* expresses 17 CU fimbriae, including ambient temperature fimbriae (ATF), mannose-resistant *Proteus*-like (MR/P), *P. mirabilis* fimbriae (PMF), *P. mirabilis* P-like pili (PMP), urothelial cell adhesin (UCA)/non-agglutinating fimbriae (NAF), Fimbria 3 (Fim3), Fim5, Fim6, Fim7, Fim8, Fim10, Fim12, Fim14, Fim15, Fim16 and Fim17, with Fim14 having no related chaperone protein [33,36,37,38]. Although not common, *P. mirabilis* also expresses type 3 fimbriae, known as mannose-resistant *Klebsiella*-like (MR/K), because they are able to agglutinate tannic acid-treated red blood cells, irrespective of the presence or absence of mannose [39]. The functional homolog of UPEC P pilus in UPPM is the UCA/NAF pilus, expressed by the *ucaABCDJ* operon. The UcaD protein is structurally similar to the UPEC F17G adhesin, and possesses a specific affinity with carbohydrate sequences (e.g., GalNAcβ1-4Gal), which are commonly present in host receptors [39]. For this reason, UCA/NAF pili are considered to play an important role in *P. mirabilis* UTIs. Adhesive structures are highly redundant, and UPPM expresses the *pmfACDEF* and the *mrpABCDEFGHJ* operons that code for PMF and MR/P fibers, respectively, mediating bacterial attachment to both kidney and bladder urothelial cells [39]. Together, these three CU fimbriae orchestrate UPPM catheter colonization and UTI pathogenesis, with the MR/P phase-variable fimbriae significantly contributing to the fitness of *P. mirabilis* in mouse models of UTIs [36]. In addition, MR/P mediates the formation of large intraluminal bacterial clusters that extend over the entire length of the bladder. This occurs immediately after bacterial adhesion to the epithelial cells, with the help of UCA and PMF fimbriae. It was demonstrated that bacterial clusters serve as the site of stone formation; bacteria enclosed in the cluster undergo phase variation, and a mixed population with MR/P-OFF and MR/P-ON are responsible for bladder stone-formation in mice [40]. Moreover, MR/P-OFF bacterial cells can disseminate from the cluster/stone, using the well-known flagella-mediated motility to easily spread the infection [40]. Moreover, several genes belonging to the CU system have been recently identified in *K. pneumoniae*, such as the *kpj* cluster encoding the CU fimbriae involved in intestinal colonization [41].

According to previous studies, more than 80% of *K. pneumoniae* strains encode type 1 and type 3 (also known as mannose-resistant *Klebsiella*-like hemagglutinins) CU fimbriae. Located in the bacterial chromosome, type 1 and 3 pili genes are organized in operons, namely *fimBEAICDFGH* and *mrkABCDF* operons, respectively [42]. Interestingly, the type 1 pilus operon is present in some strains of UPEC harboring IncX-plasmids, which are commonly present in *Enterobacteriaceae* and known to carry *Klebsiella*-derived operons [43]. In addition, Kpc fimbriae, encoded by the *kpcABCDEFG* gene cluster, and the recently discovered new CU fimbriae encoded by the *kpj* cluster, have been observed in *K. pneumoniae*, emphasizing the need for CU fimbrial redundancy of this microorganism [41,44]. Altogether, these fimbriae represent the arsenal of UPKP adhesins; type 1 pili are mainly involved in the interaction with bladder epithelial cells, whereas the others are mostly involved in the adhesion to abiotic surfaces (e.g., medical devices) and in biofilm formation [5,42,45]. It has been shown that type 3 pili together with type 1 are strictly required to cause catheter-associated UTIs (CAUTIs), a common hospital-acquired infection [45]. Most CAUTIs are polymicrobial, usually involving *K. pneumoniae* in association with *P. mirabilis* or this latter in co-infection with *E. coli*, highlighting the adoption of shared strategies to colonize and reside within human hosts [46]. Hence, targeting adhesive structures represents an excellent option to prevent and treat UTIs. 

## 4. FimH is a Highly Adapted Virulence Factor

Among the CU pili from uropathogenic members belonging to *Enterobacteriaceae*, one of the best-characterized is type 1 pili. These pili are expressed by 80% and 90% of UPKP and UPEC strains, respectively [5,47,48,49]. It has been reported that more than 95% of all *E. coli* isolates express type 1 fimbriae [50,51,52]. The type 1 pilus is 2 μm in length and 10 nm in width, and is highly represented in the bacterial surface (100–500 pili per cell) [5,47,48,49]. This pilus is defined as mannose-sensitive, because it is able to interact with the mannosylated receptors expressed by epithelial cells, particularly urothelial cells [12,19,48,53,54]. This specific function relies on the expression of the adhesin FimH located at the tip of the type 1 pilus. Type 1 pili of *K. pneumoniae* and *E. coli* are highly homologous in uropathogenic strains. However, the slight sequence variations between the FimH from *E. coli* and *K. pneumoniae* result in huge differences in the ability to colonize the urinary tract, FimH from UPEC being much more efficient in adhering to the mannosylated structure; for these reasons, it was chosen as the model for the bacteria–urothelium interaction [55]. Proper functioning of urothelium depends on the precise assemblage of highly specialized glycoproteins called uroplakins (UPs), the end products and differentiation markers of urothelial cells. On the apical surface, four major UPs are expressed by the umbrella cells lining the bladder, forming hexagonal plaques characterized by six tetramers linked by two heterodimers, UPIa/II and Ib/IIIa. UPs can be synthesized in several glycoforms; UPIa contains high-mannose N-glycans, and UPIb and IIIa carry complex N-glycans, whereas mature UPII lacks sugar moieties [56,57]. FimH binds to high-mannosylated UPIa, thereby ensuring a stable bacterial adhesion to the tissue. It is noteworthy that FimH is also responsible for biofilm formation, proliferation, and invasion of and internalization into eukaryotic cells, mediating the formation of intracellular bacterial communities (IBCs) [8,54,58]. Moreover, FimH is also able to interact with the Tamm–Horsfall soluble proteins, which are secreted by kidney cells, within the urine to exert a protective role against FimH adhesion [53,59]. Finally, CD48, types I and IV collagens, fibronectins and laminins are other receptors that can be bound by the UPEC FimH [60]. Due to the multiplicity of ligands and functions, type 1 pili armed with FimH represent a pivotal virulence factor within UPEC [5,8,19,30,61]. 

**Figure 1:**
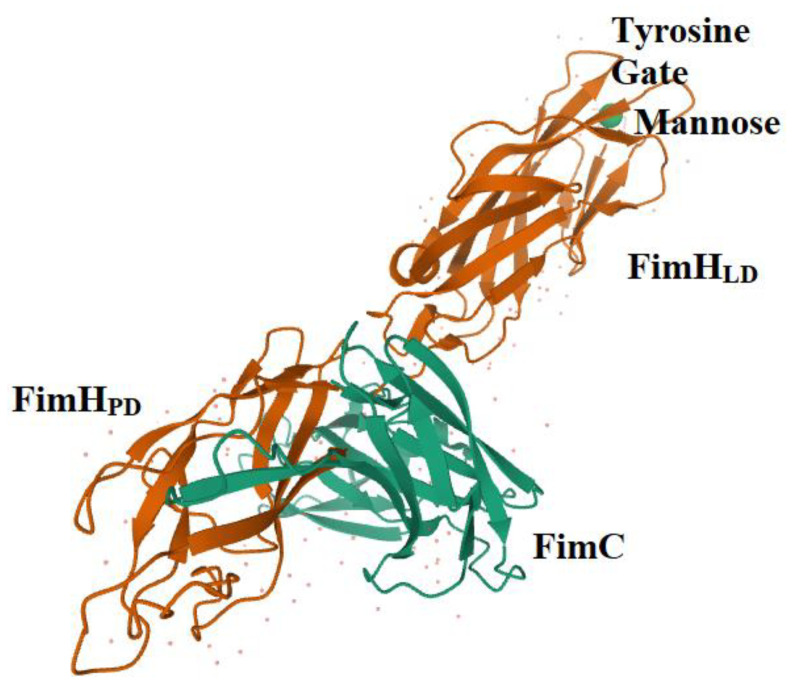
The structure of FimH co-crystallized with FimC. The FimH_PD_, FimH_LD_, Tyrosine Gate (Tyr137, Ile52 and Tyr48), molecule of mannose and FimC is shown (1KLF PDB file) [62]. FimC is a chaperone protein that does not belong to the mature fimbria.

The whole FimH adhesin is composed of 279 amino acids. The N-terminal domain (NTD) carries a lectin domain (FimH_LD_) encompassing the carbohydrate-binding domain (CBD), while the C-terminal domain (CTD) bears a pilin domain (FimH_PD_) (Figure 1) [19,54,62]. The interaction between these two domains, FimH_LD_ and FimH_PD,_ determines the conformational state of the FimH adhesin, thereby influencing the level of affinity of FimH with the related molecule/receptor/ligand [13,54]. The conformation of FimH is highly dynamic, and interdomain interactions can be influenced by different factors, including the shear stress. Normally, FimH is in the low affinity conformation, also known as T-state; however, it switches to the high affinity structure in the presence of shear forces (R-state). The mechanism by which FimH binds to the mannosylated uroplakins is known as the catch and bond mechanism, which enables bacteria to establish long-lived interactions with host cells [13,54].

**Figure 2:**
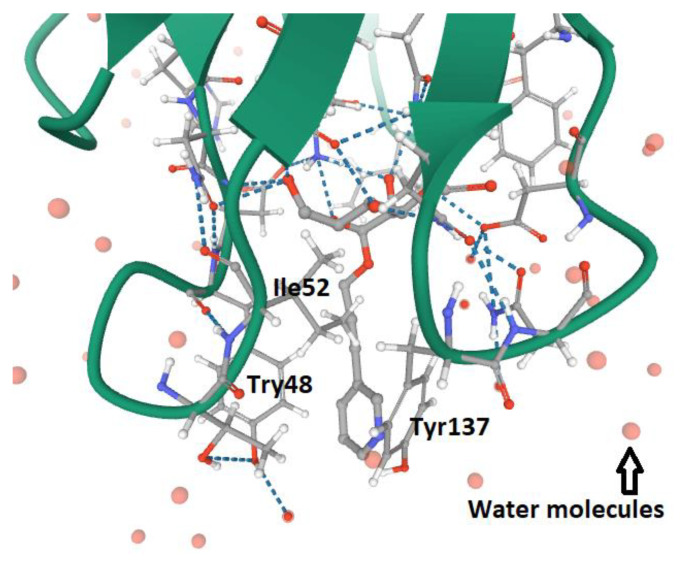
The structure of the tyrosine gate of FimH_LD_ MBP in UPEC (Tyr137, Ile52 and Tyr48). The ligand is α-D-mannoside O-linked to a propynyl pyridine (4AV4 PDB file) [64].

The CBD within FimH_LD_ is responsible for the binding to the mannosylated molecules, in that the amino acid’s composition allows the formation of the negatively charged mannose-binding pocket (MBP), explaining why amino acids encompassing the MBP are extremely conserved among UPEC strains [61,63]. The MBP is surrounded by hydrophobic amino acids, comprising Ile13, Tyr48, Ile52, Tyr137 and Phe142 [61,63,64]. The dynamic conformation of amino acid residues Tyr 48 and 137 constitutes the structure of the tyrosine gate (Figure 2) [61,63,64], which covers the hydrophobic groove of the mannose-binding site (MBS) [63,64]. 

**Figure 3:**
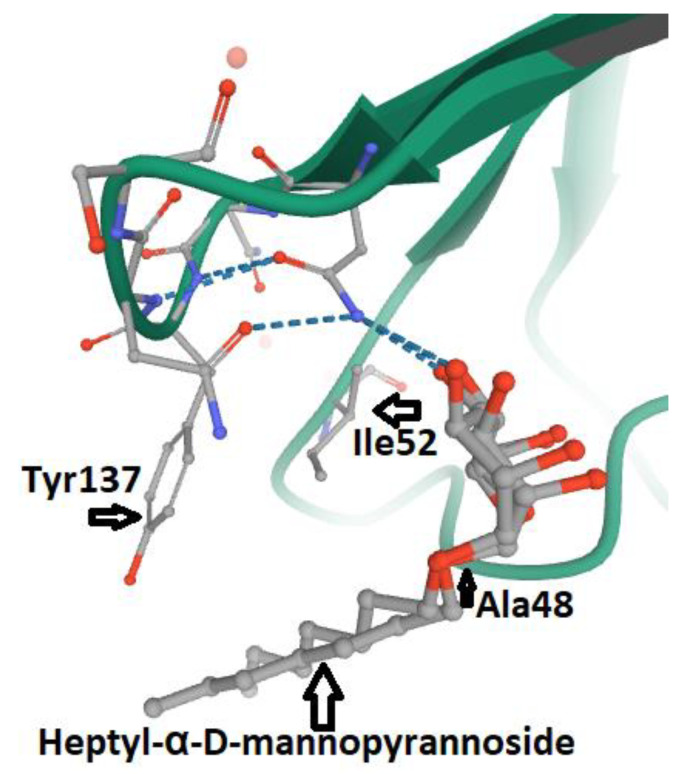
The Tyr48Ala mutation within the relaxed Tyrosine Gate of MBP in FimH_LD_ from UPEC. The linkage of Heptyl-α-D-mannopyrannoside with mutated Tyrosine Gate is shown. The stacking pattern between Tyr137, Ile52, Ala48 and Heptyl-α-D-mannopyrannoside is shown (4CA4 PDB file) [65].

The FimH_LD_ MBS consists of a hydrophobic region (including Phe142, Phe1 and Ile13), a stretch of seven polar amino acids (Asn46, Asp47, Asp54, Gln133, Asn135, Asn138 and Asp140), the tyrosine gate (Tyr137, Ile52, Tyr48) and the Thr51 amino acid residue. The affinity between ligands and the MBP of FimH can be increased by Van der Waals bonds within the hydrophobic groove [19,63]. The side chains of the Tyr molecules at positions 48 and 137 are dynamic rotamers that can define three different tyrosine gate configurations: the full open, full close and partly open gate [63,64]. Any mutation in the tyrosine gate amino acids may lead to the loss of affinity with mannosylated molecules by FimH (Figure 3) [65]. Moreover, any mutation within the amino acids of the MBP of FimH, including Phe1, Asn46, Asp47, Asp54, Gln133, Asn135, Asp140 and Phe142, results in the abrogation of the adhesin function [19,62,66].

## 5. FimH and Glycomimetics

Since FimH emerged as the most appropriate target for the development of anti-adhesive therapeutic strategies, several studies began, decades ago, to analyze the effects of FimH antagonists. Duguid and Gillis were the first authors to report mannose as an anti-adhesive substance in *E. coli* bacteria in 1957 [19,67]. Then, in 1977, the anti-adhesive activity of mannose was described in detail by Ofek, Mirelman and Sharon for *E. coli* [19,68], and later on for other uropathogens [69,70,71]. Up until now, different categories of soluble mono- and polyvalent FimH inhibitors (α-d-mannosides, and their chemically modified derivatives and glycodendrimers, respectively) have been selected, synthesized and analyzed [17,54]. Due to the huge number of available molecules, researchers in this field established standardized protocols and techniques to test FimH inhibitory activity. The relative inhibitory potency (RIP) index describes the extent of the affinity of the various FimH antagonists, compared to known synthetic mannoside derivatives such as the Methyl α-d-mannoside (MeMan), which is considered as a high-affinity molecule with regards to FimH [48,72]. The application of this method ensures the comparability of the results obtained by applying different experimental procedures. High RIP values result in the strong affinity of the analyzed molecules. For example, it has been shown that a monovalent antagonist with a high RIP value possessed the same affinity toward a variety of UPEC strains, while a polyvalent antagonist showing high RIP displayed a strain-dependent affinity. This result points out that more studies should be performed in assessing the therapeutic efficacy of polyvalent molecules [73]. However, despite their high and broad activity, monovalent glycosides, such as natural d-mannose, have no stable structures in vivo, and are rapidly hydrolyzed within the mouth, gastrointestinal tract and other organs and tissues, or they are quickly excreted from the body [18,74]. So, the application of antagonist therapeutic strategies, such as glycosidic drugs, represents a big challenge. The advances in understanding carbohydrate–protein interactions led to the development of a new class of small-molecule drugs for the treatment of several human diseases, known as glycomimetics. These molecules mimic the bioactive function of pure carbohydrates without presenting their drawbacks, such as low activity and stability [75]. Thus, specific chemical modifications represented a good approach to enhancing the bioavailability and metabolic stability of glycoside molecules [18]. Today, several glycomimetics, based on α-d-mannose derivatives, have been selected that show a strong affinity with FimH [61]. Glycosides are chemically linked to the aglycone (non-carbohydrate) portion in order to improve the glycomimetics’ affinity to FimH, as well as their stability and bioavailability. Alkyl-, aryl- biaryl-, biphenyl-, butyl- dioxocyclobutenylaminophenyl-, indolylphenyl-, methyl-, phenyl-, triazolyl-, thiazolylamine- and umbelliferyl- are the most common aglycone groups combined with monovalent α-d-mannosides [19,54,61,63,76,77]. Vice versa, the polyvalent d-mannosides include cyclodextrin-based heptyl mannosides (CD-based HMs), divalent mannosides, glycoclusters, glycodendrimers, neoglycoproteins and trivalent mannosides, which contain more than one mannose subunit [19,61,63,78]. Indeed, lectins may carry several carbohydrate-binding sites (CBS), and this property enables them to significantly increase their affinity towards sugar residues. Exploiting this feature, multivalent glycomimetics are bound by FimH with high affinity, and this multivalent effect is known as molecule avidity [79,80]. These types of modifications result in an inhibition effect on FimH a million times greater than that exerted by the d-mannose sugar [30,81,82]. 

## 6. Assays to Test FimH Antagonist Activity: Competitive and Inhibition Tests 

The affinity strength of synthetic FimH antagonists can be evaluated using two different approaches. The first one is based on competitive assays, in which the affinity of the selected antagonist is compared to a known compound with a high affinity for FimH. Several techniques were adapted to perform this competitive assay, such as Biolayer Interferometry Assay (BIA), cell-free high-throughput competitive binding assay, Differential Scanning Fluorimetry (DSF), Enzyme-Linked Immuno Sorbent Assay (ELISA), Fluorescence Polarization Assay (FPA), Isothermal Titration Calorimetry (ITC), Radioactive Labeling Assay (RLA) and Surface Plasmon Resonance (SPR) [19,54,81,82,83,84,85,86,87,88,89,90]. The second one analyzes the affinity of the antagonist by measuring the inhibition of FimH’s biological function, and includes the aggregation, biofilm inhibition, disaggregation, flow cytometry, hemagglutination inhibition and epithelial cell adherence inhibition assays [19,24,54,69,91,92,93,94]. However, both competitive and inhibition tests present some drawbacks; for example, depending on the assay performed, the RIP of the reference compounds (d-mannose and MeMan) ranges between millimolar and micromolar, making the comparison of different FimH antagonists difficult. In addition, tests with intact bacteria can be affected by the presence of FimH mutations and the different levels of FimH expression among the strains tested [86]. For this reason, more accurate, reproducible and standardized tests are needed to explore the FimH antagonist’s effectiveness.

## 7. FimH Antagonists, Biochemical Characteristics and Bioavailability

According to the type of interaction with the d-mannosylated molecules, FimH adhesin changes its conformational structure, leading to different binding affinities. As outlined above, the low affinity (T-state) conformational structure of FimH, in which the LD and PD domains are in strict contact, occurs in the absence of shear stress. Vice versa, the high affinity conformation (R-state), in which FimH_LD_ and Fim_PD_ are separated, represents the shear stress-induced allosteric regulation of its mannose-binding affinity, resulting in the strong attachment of FimH_LD_ to the host urothelial cell receptors [54]. Thus, the balance between R- and T-states regulates the capability of the bacteria to colonize the urothelial niche or to spread the infection. At the molecular level, it is known that the interactions between α-d-mannose molecules (and related derivatives) and MBP in FimH_LD_ occur in the presence of water, because water molecules support the hydrogen bonds between the hydroxyl groups of α-d-mannose molecules and the amino acid residues within MBP. Moreover, the presence of water drives the proper binding of α-anomer molecules to the MBP of FimH, increasing the affinity of the α-anomeric configuration of mannose and its derivatives with MBP [19,62,95,96]. Biochemical analyses of the interaction between FimH_LD_ and α-d-mannose revealed that mannosides with an apolar (hydrophobic) substituent are able to mimic the interactions of high-mannose glycans with the MBD of FimH [82]. For this reason, n-hexyl- and n-heptyl-modified mannosides (i.e., MeMan) have a significant high affinity towards FimH [19,97]. This hydrophobic portion of aglycone interacts with the tyrosine gate through aromatic stacking (non-covalent interaction between aromatic rings) and Van der Waals bonds [77,97,98,99]. Moreover, it was shown that glycomimetics with inhibition constants in the range of 1–20 nM can be obtained by combining the α-anomeric configurations of d-mannose [96,100]. Hence, Wellens et al. generated a set of α-d-mannosides carrying alkyl and aryl hydrophobic moieties. The determination of the crystal structure of FimH_LD_ with the eight synthesized inhibitors, together with the analyses of their thermodynamic parameters, demonstrated that the presence of alkyl and aryl groups in the aglycone can induce the increased dynamics in the tyrosine gate responsible for the proper orientation of the interacting mannosides. This dynamic behavior of the tyrosine gate could contribute to FimH’s ability to deal with less compatible high-mannose structures, while still making bacterial adhesion plausible [64]. Moreover, aromatic aglycone compounds mediate several interactions within the tyrosine gate in its hydrophobic space, increasing the affinity of the antagonist to the MBP of FimH_LD_ [80,96]. An increase in the length of alkyl chains results in the higher affinity of the molecule with the FimH_LD_ and, in particular, with the tyrosine gate area, showing that the affinity of the alkyl group with FimH adhesin is 100-fold greater than that exhibited by mannose [19,25,89]. 

**Figure 4:**
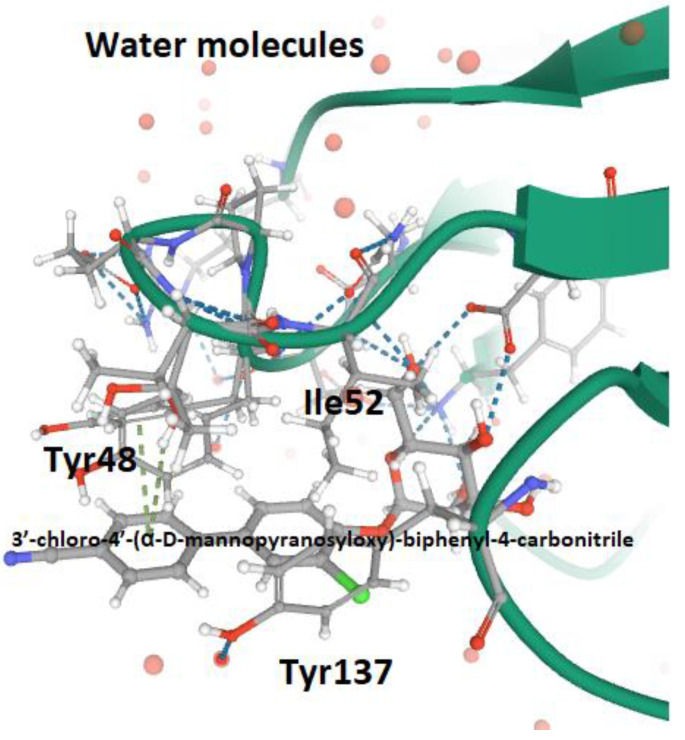
The successful linkage between Tyrosine Gate (Fim_LD_ MBP) and the bioisostere of 3′-chloro-4′-(α-d-mannopyranosyloxy)-biphenyl-4-carbonitrile (4CST PDB file) [47].

It has been shown that O- and C-linked α-d-mannosides with hydrophobic and aryl substituents are potent *E. coli* FimH antagonists, having an affinity in the same range as that of nanomolar [101]. Indeed, the conformation and lipophilicity of aglycone moieties, their position with respect to the core sugar structure and the type of chemical group determine the RIP of antagonist molecules [61,80]. Para-substituted biphenyl derivatives were shown to be particularly appealing, owing to their numerous favorable binding interactions within the tyrosine gate. Thus, the structural and functional analyses of a series of O-, C-, and S-linked mannoside derivatives, incorporating the 1,1′-biphenyl pharmacophore and diverse aglycone atoms, demonstrated the suitability of these antagonists, establishing the possibility of further exploring these chemically modified mannosides [101,102]. Furthermore, it was shown that the biphenyl group linked to mannosides can be efficiently absorbed if orally administered [54,88]. Indeed, these mannosides show increased metabolic stability, bioavailability and intestinal permeability in in vivo pharmacokinetic studies, thereby recommending them for preclinical evaluation [83]. In addition, the reabsorption of biphenyl groups by renal tubuli results in stable and regular excretion into urine, leading to their high availability in the site of infection [54,88,103]. It was also demonstrated that 3′-chloro-4′-(α-d-mannopyranosyloxy) biphenyl-4-carbonitriler (Figure 4), synthesized using the bioisostere approach, is a highly effective FimH antagonist, also presenting optimal pharmacokinetic characteristics, such as proper solubility, low toxicity, intestinal permeability and renal excretion in mouse models [47]. Moreover, its oral application reduced the bacterial load in the bladder by almost 1000-fold 3 h after infection, highlighting its therapeutic potential [47].

The polyvalent adhesin inhibitors (carbohydrate dendrimers) were designed to better mimic the interaction of FimH with high-mannose eukaryotic receptors [61]. The affinity, avidity and selectivity of mannosylated glycodendrimers are strengthened throughout by the presence of several mannose residues in the molecule; the so-called “cluster effect” [80,97,104]. Despite their higher affinity with the MBP of FimH, mannosylated glycodendrimers are large-size polar molecules, and these chemical properties reduce their absorption in the gastrointestinal tract, affecting their oral usage [61]. 

Apart from chemically synthesized mannose-based molecules, natural compounds, including cranberry and its derivatives, such as myricetin, cranberry extract standardized in proanthocyanidins (PACs) and PAC-derived polyphenol metabolites, have anti-adhesive effects on UPEC [53]. The mechanism by which these compounds exert their anti-adhesive activity is not totally understood yet. The complex molecular composition of these natural extracts can influence the establishment of the infection at different levels, acting on both bacteria and human physiology. Several investigations showed that PACs efficiently block the P fimbriae [105,106,107]. Vice versa, it was indicated that PAC-metabolites could be responsible for anti-adhesive effects on FimH [53]. Moreover, it was also suggested that cranberry induces the expression/secretion of the Tamm–Horsfall proteins by the kidney, thereby leading to its accumulation in the bladder. Thus, the interaction between UPEC FimH and the mannosylated Tamm–Horsfall glycoproteins causes bacterial release within the urine flux [108]. As such, cranberry-based supplements represent a source of natural compounds that are biochemically active against UPEC, which deserves further investigation.

## 8. Conclusions

UTIs and CAUTIs are becoming increasingly important threats to human health, due to the rise of MDR uropathogens. The anti-adhesive strategy emerged as a relevant alternative therapeutic approach, targeting uropathogen virulence traits. FimH is one of the most-studied adhesins expressed by UPEC strains because it is a key determinant of urovirulence. Indeed, FimH interacts with high-mannosylated uroplakins, enabling the bacterium to stably adhere to bladder cells and, eventually, to be internalized. Therefore, its chemical and functional characteristics have been, and still are, used to find molecules to inhibit bacterial adhesion. Carbohydrate-based drugs, known as glycomimetics, offer a powerful opportunity to block FimH by mimicking the structure and function of native carbohydrate. Up to now, a huge number of mannose-based glycomimetics have been synthesized and tested for their affinity and efficacy in hindering the FimH engagement of mannosylated receptors. Due to its availability in vegetables and fruits, natural d-mannose is already used as a supplement for the prevention and treatment of rUTIs, or in combination with antibiotics, having shown its efficacy in different clinical studies [109,110] (https://clinicaltrials.gov/ct2/show/results/NCT01808755). Thus, numerous molecules operating on the same mode of action as the d-mannose have been developed and improved. In this review, we presented the current advances in available d-mannose derivatives and glycomimetics. These molecules represent a promising, valuable, effective, feasible and cost-effective approach to the treatment of UTIs, especially those caused by MDR UPEC, requiring urgent clinical trials.

## Abbreviations

AMR	antimicrobial resistance
ATF	ambient temperature fimbria
BIA	biolayer interferometry assay
CAUTIs	catheter-associated UTIs
CBD	carbohydrate binding domain
CBS	carbohydrate-binding site
CD-based HMs	cyclodextrin-based heptyl mannosides
CTD	C-terminal domain
CU	chaperon-usher
DSF	differential scanning fluorimetry
ECDC	European Center of Diseases Prevention and Control
ELISA	enzyme-linked immuno sorbent assay
Fim3	Fimbria 3
FPA	fluorescence polarization assay
IBCs	intracellular bacterial communities
ITC	isothermal titration calorimetry
MBP	mannose-binding pocket
MBS	mannose-binding site
MDR	multi-drug-resistant
MeMan	Methyl α-d-mannoside
MR/P	mannose-resistant *Proteus*-like
MR/K	mannose-resistant *Klebsiella*-like
NAF	non-agglutinating fimbria
NTD	N-terminal domain
PACs	proanthocyanidins
*pap*	pyelonephritis-associated pili
PDR	pan-drug-resistant
PMF	*P. mirabilis* fimbria
PMP	*P. mirabilis* P-like pili
RIP	relative inhibitory potency
RLA	radioactive labeling assay
rUTI	recurrent UTI
SPR	surface plasmon resonance
UCA	urothelial cell adhesin
UTI	urinary tract infection
UPEC	uropathogenic *Escherichia coli*
UPKP	uropathogenic *Klebsiella pneumoniae*
UPPM	uropathogenic *Proteus mirabilis*
UPs	uroplakins

## References

[1] Flores-Mireles A.L., Walker J.N., Caparon M., Hultgren S.J. (2015). Urinary tract infections: Epidemiology, mechanisms of infection and treatment options. Nat. Rev. Microbiol..

[2] Behzadi P., Behzadi E., Pawlak-Adamska E.A. (2019). Urinary tract infections (UTIs) or genital tract infections (GTIs)? It’s the diagnostics that count. GMS Hyg. Infect. Control.

[3] Chockalingam A., Stewart S., Xu L., Gandhi A., Matta M.K., Patel V., Sacks L., Rouse R. (2019). Evaluation of immunocompetent urinary tract infected Balb/C mouse model for the study of antibiotic resistance development using *Escherichia Coli* CFT073 infection. Antibiotics.

[4] Issakhanian L., Behzadi P. (2019). Antimicrobial agents and urinary tract infections. Curr. Pharm. Des..

[5] Behzadi P. (2020). Classical chaperone-usher (CU) adhesive fimbriome: Uropathogenic Escherichia coli (UPEC) and urinary tract infections (UTIs). Folia Microbiol. (Praha).

[6] Hozzari A., Behzadi P., Kerishchi Khiabani P., Sholeh M., Sabokroo N. (2020). Clinical cases, drug resistance, and virulence genes profiling in Uropathogenic Escherichia coli. J. Appl. Genet..

[7] Momtaz H., Karimian A., Madani M., Safarpoor Dehkordi F., Ranjbar R., Sarshar M., Souod N. (2013). Uropathogenic Escherichia coli in Iran: Serogroup distributions, virulence factors and antimicrobial resistance properties. Ann. Clin. Microbiol. Antimicrob..

[8] Jahandeh N., Ranjbar R., Behzadi P., Behzadi E. (2015). Uropathogenic Escherichia coli virulence genes: Invaluable approaches for designing DNA microarray probes. Cent. Eur. J. Urol..

[9] Behzadi P., Najafi A., Behzadi E., Ranjbar R. (2016). Microarray long oligo probe designing for Escherichia coli: An in-silico DNA marker extraction. Cent. Eur. J. Urol..

[10] Scribano D., Sarshar M., Prezioso C., Lucarelli M., Angeloni A., Zagaglia C., Palamara A.T., Ambrosi C. (2020). D-Mannose Treatment neither Affects Uropathogenic *Escherichia coli* Properties nor induces stable fimh modifications. Molecules..

[11] Umpiérrez A., Scavone P., Romanin D., Marqués J.M., Chabalgoity J.A., Rumbo M., Zunino P. (2013). Innate immune responses to proteus mirabilis flagellin in the urinary tract. Microbes Infect..

[12] Behzadi E., Behzadi P. (2016). The role of toll-like receptors (TLRs) in urinary tract infections (UTIs). Cent. Eur. J. Urol..

[13] Terlizzi M.E., Gribaudo G., Maffei M.E. (2017). UroPathogenic *Escherichia coli* (UPEC) infections: Virulence factors, bladder responses, antibiotic, and non-antibiotic antimicrobial strategies. Front. Microbiol..

[14] Behzadi P., Urbán E., Matuz M., Benkő R., Gajdács M. (2020). The role of gram-negative bacteria in urinary tract infections: Current concepts and therapeutic options. Adv. Exp. Med. Biol..

[15] Schaffer J.N., Pearson M.M. (2015). Proteus mirabilis and urinary tract infections. Microbiol. Spectr..

[16] Wyres K.L., Lam M., Holt K.E. (2020). Population genomics of klebsiella pneumoniae. Nat. Rev. Microbiol..

[17] Psonis J.J., Thanassi D.G. (2019). Therapeutic approaches targeting the assembly and function of chaperone-usher pili. EcoSal Plus.

[18] Mydock-McGrane L., Cusumano Z., Han Z., Binkley J., Kostakioti M., Hannan T., Pinkner J.S., Klein R., Kalas V., Crowley J. (2016). Antivirulence c-mannosides as antibiotic-sparing, oral therapeutics for urinary tract infections. J. Med. Chem..

[19] Mydock-McGrane L.K., Hannan T.J., Janetka J.W. (2017). Rational design strategies for FimH antagonists: New drugs on the horizon for urinary tract infection and Crohn’s disease. Expert Opin. Drug Discov..

[20] Maddirala A.R., Klein R., Pinkner J.S., Kalas V., Hultgren S.J., Janetka J.W. (2019). Biphenyl Gal and GalNAc FmlH lectin antagonists of uropathogenic E. coli (UPEC): Optimization through iterative rational drug design. J. Med. Chem..

[21] Kalograiaki I., Abellán-Flos M., Fernández L.Á., Menéndez M., Vincent S.P., Solís D. (2018). Direct evaluation of live uropathogenic Escherichia coli adhesion and efficiency of antiadhesive compounds using a simple microarray approach. Anal. Chem..

[22] Hernando-Amado S., Coque T.M., Baquero F., Martínez J.L. (2019). Defining and combating antibiotic resistance from one health and global health perspectives. Nat. Microbiol..

[23] Thänert R., Reske K.A., Hink T., Wallace M.A., Wang B., Schwartz D.J., Seiler S., Cass C., Burnham C.A., Dubberke E.R. (2019). Comparative genomics of antibiotic-resistant uropathogens implicates three routes for recurrence of urinary tract infections. mBio.

[24] Klein R.D., Hultgren S.J. (2020). Urinary tract infections: Microbial pathogenesis, host-pathogen interactions and new treatment strategies. Nat. Rev. Microbiol..

[25] Asadi A., Razavi S., Talebi M., Gholami M. (2019). A review on anti-adhesion therapies of bacterial diseases. Infection.

[26] Patel S., Mathivanan N., Goyal A. (2017). Bacterial adhesins, the pathogenic weapons to trick host defense arsenal. Biomed. Pharmacother..

[27] Solanki V., Tiwari M., Tiwari V. (2018). Host-bacteria interaction and adhesin study for development of therapeutics. Int. J. Biol. Macromol..

[28] Berne C., Ducret A., Hardy G.G., Brun Y.V. (2015). adhesins involved in attachment to abiotic surfaces by gram-negative bacteria. Microbiol. Spectr..

[29] Behzadi P., Urbán E., Gajdács M. (2020). Association between biofilm-production and antibiotic resistance in uropathogenic *Escherichia coli* (UPEC): An in vitro study. Diseases.

[30] Zalewska-Piątek B.M., Piątek R.J. (2019). Alternative treatment approaches of urinary tract infections caused by uropathogenic Escherichia coli strains. Acta Biochim. Pol..

[31] Wurpel D.J., Beatson S.A., Totsika M., Petty N.K., Schembri M.A. (2013). Chaperone-Usher fimbriae of Escherichia coli. PLoS ONE.

[32] Wurpel D.J., Totsika M., Allsopp L.P., Webb R.I., Moriel D.G., Schembri M.A. (2016). Comparative proteomics of uropathogenic Escherichia coli during growth in human urine identify UCA-like (UCL) fimbriae as an adherence factor involved in biofilm formation and binding to uroepithelial cells. J. Proteom..

[33] Kuan L., Schaffer J.N., Zouzias C.D., Pearson M.M. (2014). Characterization of 17 chaperone-usher fimbriae encoded by Proteus mirabilis reveals strong conservation. J. Med. Microbiol..

[34] Dodson K.W., Pinkner J.S., Rose T., Magnusson G., Hultgren S.J., Waksman G. (2001). Structural basis of the interaction of the pyelonephritic E. coli adhesin to its human kidney receptor. Cell.

[35] Lillington J., Geibel S., Waksman G. (2014). Biogenesis and adhesion of type 1 and P pili. Biochim. Biophys. Acta.

[36] Pearson M.M., Sebaihia M., Churcher C., Quail M.A., Seshasayee A.S., Luscombe N.M., Abdellah Z., Arrosmith C., Atkin B., Chillingworth T. (2008). Complete genome sequence of uropathogenic Proteus mirabilis, a master of both adherence and motility. J. Bacteriol..

[37] Jiang W., Ubhayasekera W., Pearson M.M., Knight S.D. (2018). Structures of two fimbrial adhesins, AtfE and UcaD, from the uropathogen Proteus mirabilis. Acta Crystallogr. D Struct. Biol..

[38] Armbruster C.E., Mobley H., Pearson M.M. (2018). Pathogenesis of Proteus mirabilis infection. EcoSal Plus.

[39] Rocha S.P., Pelayo J.S., Elias W.P. (2007). Fimbriae of uropathogenic Proteus mirabilis. FEMS Immunol. Med. Microbiol..

[40] Norsworthy A.N., Pearson M.M. (2017). From catheter to kidney stone: The uropathogenic lifestyle of Proteus mirabilis. Trends Microbiol..

[41] Khater F., Balestrino D., Charbonnel N., Dufayard J.F., Brisse S., Forestier C. (2015). In silico analysis of usher encoding genes in Klebsiella pneumoniae and characterization of their role in adhesion and colonization. PLoS ONE.

[42] Wilksch J.J., Yang J., Clements A., Gabbe J.L., Short K.R., Cao H., Cavaliere R., James C.E., Whitchurch C.B., Schembri M.A. (2011). MrkH, a novel c-di-GMP-dependent transcriptional activator, controls Klebsiella pneumoniae biofilm formation by regulating type 3 fimbriae expression. PLOS Pathog..

[43] Burmølle M., Norman A., Sørensen S.J., Hansen L.H. (2012). Sequencing of IncX-plasmids suggests ubiquity of mobile forms of a biofilm-promoting gene cassette recruited from Klebsiella pneumoniae. PLoS ONE.

[44] Wu C.C., Huang Y.J., Fung C.P., Peng H.L. (2010). Regulation of the Klebsiella pneumoniae Kpc fimbriae by the site-specific recombinase KpcI. Microbiology.

[45] Martin R.M., Bachman M.A. (2018). Colonization, infection, and the accessory genome of Klebsiella pneumoniae. Front. Cell. Infect. Microbiol..

[46] Kotaskova I., Obrucova H., Malisova B., Videnska P., Zwinsova B., Peroutkova T., Dvorackova M., Kumstat P., Trojan P., Ruzicka F. (2019). Molecular techniques complement culture-based assessment of bacteria composition in mixed biofilms of urinary tract catheter-related samples. Front. Microbiol..

[47] Kleeb S., Pang L., Mayer K., Eris D., Sigl A., Preston R.C., Zihlmann P., Sharpe T., Jakob R.P., Abgottspon D. (2015). FimH antagonists: Bioisosteres to improve the in vitro and in vivo PK/PD profile. J. Med. Chem..

[48] Hartmann M., Papavlassopoulos H., Chandrasekaran V., Grabosch C., Beiroth F., Lindhorst T.K., Röhl C. (2012). Inhibition of bacterial adhesion to live human cells: Activity and cytotoxicity of synthetic mannosides. FEBS Lett..

[49] Stahlhut S.G., Struve C., Krogfelt K.A. (2012). Klebsiella pneumoniae type 3 fimbriae agglutinate yeast in a mannose-resistant manner. J. Med. Microbiol..

[50] Sokurenko E.V., Chesnokova V., Dykhuizen D.E., Ofek I., Wu X.R., Krogfelt K.A., Struve C., Schembri M.A., Hasty D.L. (1998). Pathogenic adaptation of Escherichia coli by natural variation of the FimH adhesin. Proc. Nat.l Acad. Sci. USA.

[51] Sarshar M., Scribano D., Marazzato M., Ambrosi C., Aprea M.R., Aleandri M., Pronio A., Longhi C., Nicoletti M., Zagaglia C. (2017). Genetic diversity, phylogroup distribution and virulence gene profile of pks positive Escherichia coli colonizing human intestinal polyps. Microb. Pathog..

[52] Ambrosi C., Sarshar M., Aprea M.R., Pompilio A., Di Bonaventura G., Strati F., Pronio A., Nicoletti M., Zagaglia C., Palamara A.T. (2019). Colonic adenoma-associated Escherichia coli express specific phenotypes. Microbes Infect..

[53] Rafsanjany N., Senker J., Brandt S., Dobrindt U., Hensel A. (2015). In vivo consumption of cranberry exerts ex vivo antiadhesive activity against FimH-Dominated uropathogenic Escherichia coli: A combined in vivo, ex vivo, and in vitro study of an extract from vaccinium macrocarpon. J. Agric. Food Chem..

[54] Mayer K., Eris D., Schwardt O., Sager C.P., Rabbani S., Kleeb S., Ernst B. (2017). Urinary tract infection: Which conformation of the bacterial lectin FimH is therapeutically relevant?. J. Med. Chem..

[55] Rosen D.A., Pinkner J.S., Walker J.N., Elam J.S., Jones J.M., Hultgren S.J. (2008). Molecular variations in Klebsiella pneumoniae and Escherichia coli FimH affect function and pathogenesis in the urinary tract. Infect. Immun..

[56] Zhou G., Mo W.J., Sebbel P., Min G., Neubert T.A., Glockshuber R., Wu X.R., Sun T.T., Kong X.P. (2001). Uroplakin Ia is the urothelial receptor for uropathogenic Escherichia coli: Evidence from in vitro FimH binding. J. Cell Sci..

[57] Kątnik-Prastowska I., Lis J., Matejuk A. (2014). glycosylation of uroplakins. Implications for bladder physiopathology. Glycoconj. J..

[58] Lewis A.J., Richards A.C., Mulvey M.A. (2016). Invasion of host cells and tissues by uropathogenic bacteria. Microbiol. Spectr..

[59] Bates J.M., Raffi H.M., Prasadan K., Mascarenhas R., Laszik Z., Maeda N., Hultgren S.J., Kumar S. (2004). Tamm-Horsfall protein knockout mice are more prone to urinary tract infection: Rapid communication. Kidney Int..

[60] Eto D.S., Jones T.A., Sundsbak J.L., Mulvey M.A. (2007). Integrin-Mediated host cell invasion by type 1-piliated uropathogenic Escherichia coli. PLoS Pathog..

[61] Ribić R., Meštrović T., Neuberg M., Kozina G. (2018). Effective anti-adhesives of uropathogenic Escherichia coli. Acta Pharm..

[62] Hung C.S., Bouckaert J., Hung D., Pinkner J., Widberg C., DeFusco A., Auguste C.G., Strouse R., Langermann S., Waksman G. (2002). Structural basis of tropism of Escherichia coli to the bladder during urinary tract infection. Mol. Microbiol..

[63] Mydock-McGrane L.K., Cusumano Z.T., Janetka J.W. (2016). Mannose-Derived FimH antagonists: A promising anti-virulence therapeutic strategy for urinary tract infections and Crohn’s disease. Expert Opin. Ther. Pat..

[64] Wellens A., Lahmann M., Touaibia M., Vaucher J., Oscarson S., Roy R., Remaut H., Bouckaert J. (2012). The tyrosine gate as a potential entropic lever in the receptor-binding site of the bacterial adhesin FimH. Biochemistry.

[65] Rabbani S., Krammer E.M., Roos G., Zalewski A., Preston R., Eid S., Zihlmann P., Prévost M., Lensink M.F., Thompson A. (2017). Mutation of Tyr137 of the universal *Escherichia coli* fimbrial adhesin FimH relaxes the tyrosine gate prior to mannose binding. IUCr J..

[66] Chen S.L., Hung C.S., Pinkner J.S., Walker J.N., Cusumano C.K., Li Z., Bouckaert J., Gordon J.I., Hultgren S.J. (2009). Positive selection identifies an in vivo role for FimH during urinary tract infection in addition to mannose binding. Proc. Natl. Acad. Sci. USA.

[67] Duguid J.P., Gillies R.R. (1957). Fimbriæ and adhesive properties in dysentery bacilli. J. Pathol. Bacteriol..

[68] Ofek I., Mirelman D., Sharon N. (1977). Adherence of Escherichia coli to human mucosal cells mediated by mannose receptors. Nature.

[69] Firon N., Ofek I., Sharon N. (1982). Interaction of mannose-containing oligosaccharides with the fimbrial lectin of Escherichia coli. Biochem. Biophys. Res. Commun..

[70] Firon N., Ofek I., Sharon N. (1983). Carbohydrate specificity of the surface lectins of Escherichia coli, Klebsiella pneumoniae, and Salmonella typhimurium. Carbohydr. Res..

[71] Neeser J.R., Koellreutter B., Wuersch P. (1986). Oligomannoside-type glycopeptides inhibiting adhesion of Escherichia coli strains mediated by type 1 pili: Preparation of potent inhibitors from plant glycoproteins. Infect. Immun..

[72] Koliwer-Brandl H., Siegert N., Umus K., Kelm A., Tolkach A., Kulozik U., Kuballa J., Cartellieri S., Kelm S. (2011). Lectin inhibition assays for the analysis of bioactive milk sialoglycoconjugates. Int. Dairy J..

[73] Chalopin T., Brissonnet Y., Sivignon A., Deniaud D., Cremet L., Barnich N., Bouckaert J., Gouin S.G. (2015). Inhibition profiles of mono- and polyvalent FimH antagonists against 10 different Escherichia coli strains. Org. Biomol. Chem..

[74] Sattin S., Bernardi A. (2016). Glycoconjugates and glycomimetics as microbial anti-adhesives. Trends Biotechnol..

[75] Ernst B., Magnani J.L. (2009). From carbohydrate leads to glycomimetic drugs. Nat. Rev. Drug Discov..

[76] Firon N., Ashkenazi S., Mirelman D., Ofek I., Sharon N. (1987). Aromatic alpha-glycosides of mannose are powerful inhibitors of the adherence of type 1 fimbriated Escherichia coli to yeast and intestinal epithelial cells. Infect. Immun..

[77] Vanwetswinkel S., Volkov A.N., Sterckx Y.G., Garcia-Pino A., Buts L., Vranken W.F., Bouckaert J., Roy R., Wyns L., van Nuland N.A. (2014). Study of the structural and dynamic effects in the FimH adhesin upon α-d-heptyl mannose binding. J. Med. Chem..

[78] Chabre Y.M., Roy R. (2013). Multivalent glycoconjugate syntheses and applications using aromatic scaffolds. Chem. Soc. Rev..

[79] Lee Y.C., Lee R.T. (1995). Carbohydrate-protein interactions: Basis of glycobiology. Acc. Chem. Res..

[80] Hartmann M., Lindhorst T.K. (2011). The bacterial lectin FimH, a target for drug discovery-carbohydrate inhibitors of type 1 fimbriae-mediated bacterial adhesion. Eur. J. Org. Chem..

[81] Bouckaert J., Mackenzie J., de Paz J.L., Chipwaza B., Choudhury D., Zavialov A., Mannerstedt K., Anderson J., Piérard D., Wyns L. (2006). The affinity of the FimH fimbrial adhesin is receptor-driven and quasi-independent of Escherichia coli pathotypes. Mol. Microbiol..

[82] Han Z., Pinkner J.S., Ford B., Obermann R., Nolan W., Wildman S.A., Hobbs D., Ellenberger T., Cusumano C.K., Hultgren S.J. (2010). Structure-Based drug design and optimization of mannoside bacterial FimH antagonists. J. Med. Chem..

[83] Han Z., Pinkner J.S., Ford B., Chorell E., Crowley J.M., Cusumano C.K., Campbell S., Henderson J.P., Hultgren S.J., Janetka J.W. (2012). Lead optimization studies on FimH antagonists: Discovery of potent and orally bioavailable ortho-substituted biphenyl mannosides. J. Med. Chem..

[84] Scharenberg M., Schwardt O., Rabbani S., Ernst B. (2012). Target selectivity of FimH antagonists. J. Med. Chem..

[85] Tomašić T., Rabbani S., Gobec M., Mlinarič-Raščan I., Podlipnik Č., Ernst B., Anderluh M. (2014). Branched α-D-mannopyranosides: A new class of potent FimH antagonists. Med. Chem. Commun..

[86] Rabbani S., Jiang X., Schwardt O., Ernst B. (2010). Expression of the carbohydrate recognition domain of FimH and development of a competitive binding assay. Anal. Biochem..

[87] Pang L., Kleeb S., Lemme K., Rabbani S., Scharenberg M., Zalewski A., Schädler F., Schwardt O., Ernst B. (2012). FimH antagonists: Structure-Activity and structure-property relationships for biphenyl α-D-mannopyranosides. Chem. Med. Chem..

[88] Klein T., Abgottspon D., Wittwer M., Rabbani S., Herold J., Jiang X., Kleeb S., Lüthi C., Scharenberg M., Bezençon J. (2010). FimH antagonists for the oral treatment of urinary tract infections: From design and synthesis to in vitro and in vivo evaluation. J. Med. Chem..

[89] Bouckaert J., Berglund J., Schembri M., De Genst E., Cools L., Wuhrer M., Hung C.S., Pinkner J., Slättegård R., Zavialov A. (2005). Receptor binding studies disclose a novel class of high-affinity inhibitors of the Escherichia coli FimH adhesin. Mol. Microbiol..

[90] Scharenberg M., Jiang X., Pang L., Navarra G., Rabbani S., Binder F., Schwardt O., Ernst B. (2014). Kinetic properties of carbohydrate-lectin interactions: FimH antagonists. Chem. Med. Chem..

[91] Abgottspon D., Rölli G., Hosch L., Steinhuber A., Jiang X., Schwardt O., Cutting B., Smiesko M., Jenal U., Ernst B. (2010). Development of an aggregation assay to screen FimH antagonists. J. Microbiol. Methods.

[92] Scharenberg M., Abgottspon D., Cicek E., Jiang X., Schwardt O., Rabbani S., Ernst B. (2011). A flow cytometry-based assay for screening FimH antagonists. Assay Drug Dev. Technol..

[93] Hultgren S.J., Schwan W.R., Schaeffer A.J., Duncan J.L. (1986). Regulation of production of type 1 pili among urinary tract isolates of Escherichia coli. Infect. Immun..

[94] Wellens A., Garofalo C., Nguyen H., Van Gerven N., Slättegård R., Hernalsteens J.P., Wyns L., Oscarson S., De Greve H., Hultgren S. (2008). Intervening with urinary tract infections using anti-adhesives based on the crystal structure of the FimH-oligomannose-3 complex. PLoS ONE.

[95] Schönemann W., Lindegger M., Rabbani S., Zihlmann P., Schwardt O., Ernst B. (2017). 2-C-Branched mannosides as a novel family of FimH antagonists-synthesis and biological evaluation. Perspect. Sci..

[96] Ribić R., Meštrović T., Neuberg M., Kozina G. (2019). Proposed dual antagonist approach for the prevention and treatment of urinary tract infections caused by uropathogenic Escherichia coli. Med. Hypotheses.

[97] Sehad C., Shiao T.C., Sallam L.M., Azzouz A., Roy R. (2018). Effect of dendrimer generation and aglyconic linkers on the binding properties of mannosylated dendrimers prepared by a combined convergent and onion peel approach. Molecules.

[98] Touaibia M., Krammer E.M., Shiao T.C., Yamakawa N., Wang Q., Glinschert A., Papadopoulos A., Mousavifar L., Maes E., Oscarson S. (2017). Sites for dynamic protein-carbohydrate interactions of O- and C-Linked mannosides on the E. coli FimH adhesin. Molecules.

[99] Kalas V., Hibbing M.E., Maddirala A.R., Chugani R., Pinkner J.S., Mydock-McGrane L.K., Conover M.S., Janetka J.W., Hultgren S.J. (2018). Structure-Based discovery of glycomimetic FmlH ligands as inhibitors of bacterial adhesion during urinary tract infection. Proc. Natl. Acad. Sci. USA.

[100] Johnson B.K., Abramovitch R.B. (2017). Small molecules that sabotage bacterial virulence. Trends Pharmacol. Sci..

[101] Mousavifar L., Vergoten G., Charron G., Roy R. (2019). Comparative study of aryl *O*-, *C*-, and *S*-mannopyranosides as potential adhesion inhibitors toward uropathogenic *E. coli* FimH. Molecules.

[102] Mousavifar L., Touaibia M., Roy R. (2018). Development of mannopyranoside therapeutics against adherent-invasive Escherichia coli infections. Acc. Chem. Res..

[103] Schwardt O., Rabbani S., Hartmann M., Abgottspon D., Wittwer M., Kleeb S., Zalewski A., Smieško M., Cutting B., Ernst B. (2011). Design, synthesis and biological evaluation of mannosyl triazoles as FimH antagonists. Bioorg. Med. Chem..

[104] Heidecke C.D., Lindhorst T.K. (2007). Iterative synthesis of spacered glycodendrons as oligomannoside mimetics and evaluation of their antiadhesive properties. Chemistry.

[105] Gupta K., Chou M.Y., Howell A., Wobbe C., Grady R., Stapleton A.E. (2007). Cranberry products inhibit adherence of p-fimbriated Escherichia coli to primary cultured bladder and vaginal epithelial cells. J. Urol..

[106] Hisano M., Bruschini H., Nicodemo A.C., Srougi M. (2012). Cranberries and lower urinary tract infection prevention. Clinics (Sao Paulo).

[107] Nicolosi D., Tempera G., Genovese C., Furneri P.M. (2014). Anti-Adhesion activity of A2-type proanthocyanidins (a Cranberry Major Component) on uropathogenic E. coli and P. mirabilis Strains. Antibiotics.

[108] Scharf B., Sendker J., Dobrindt U., Hensel A. (2019). Influence of cranberry extract on tamm-horsfall protein in human urine and its antiadhesive activity against uropathogenic Escherichia coli. Planta Med..

[109] Domenici L., Monti M., Bracchi C., Giorgini M., Colagiovanni V., Muzii L., Benedetti Panici P. (2016). D-mannose: A promising support for acute urinary tract infections in women. A pilot study. Eur. Rev. Med. Pharmacol. Sci..

[110] Genovese C., Davinelli S., Mangano K., Tempera G., Nicolosi D., Corsello S., Vergalito F., Tartaglia E., Scapagnini G., Di Marco R. (2018). Effects of a new combination of plant extracts plus d-mannose for the management of uncomplicated recurrent urinary tract infections. J. Chemother..

